# Medical and Physical Intelligence

**Published:** 1822-06

**Authors:** 


### Medical and Physical Intelligence.

### 1. *Adhesion of the Labia Pudendi in a Negro obstructing Delivery.*

Mr. Thomas Geoghagen had, amongst his negroes in Jamaica, a woman named Avis, about 27 years of age, of the Eboe nation, who, on declaring herself in an advanced state of pregnancy, asserted at the same time that she could not be delivered, as there was no external passage, and therefore expected that she should die in consequence. There was found, on examination, to be a cicatrix extending from the mons veneris to within an inch of the anus, where there existed a small orifice barely sufficient for the introduction of a small female catheter, through which orifice the urine and menses exuded. This cicatrix appeared to have been the consequence of an operation which had been performed upon her in her native country when a child, and which she said was commonly practised with the female offspring of families of rank, as a security against improper connexions. The adhesion being removed by an incision with a sharp pointed bistoury, the delivery was easily accomplished, and future adhesion prevented by interposing a bougie till the wound of the labie on each side had healed.—*American Jour.*

### 2. *Inhalations.*

With such views, sulphuric ether is strongly recommended, and we are told, by very respectable authority, that its powers are improved by several substances which are soluble in it. Cicuta is particularly praised, half a drachm of which is to be digested in an ounce of ether, for several days, so as to form a saturated tincture. Of this, two or three tea-spoonsful are to be put into a wine glass, to be held up to the mouth, and inspired till the whole is consumed. My knowledge of this remedy enables me to speak confidently of its utility. I have tried it often, in dyspnœa from different causes, and generally with advantage. It is very useful, as was originally suggested, in consumption, and especially if it be repeated several times in the day.

But, perhaps, a still more valuable remedy of this sort I have derived from Dr. Physick. It consists of a tea-spoonful of Hoffman's anodyne liquor, and another of laudanum, which mixture is to be breathed for half an hour or more at a time.

In the forming stage of catarrh, in obstinate coryza, and in hoarseness of recent or long standing, its effects are most striking and decisive. The principle on which it acts, is obvious. The parts are here more or less inflamed, which state is relieved by the *counter agency* of the fumes coming in contact with them.—*Philadelphia Jour.*

3. *Cure of a Case of Paralysis by Lightning.*

M. Samuel Leffers, of Carteret County, in North Carolina, had been seized with a paralytic affection which fixed itself on the face, and principally on the eyes. As he was walking in his chamber, a flash of lightning struck him down senseless ; he came to himself at the end of twenty minutes, but he did not recover perfectly the use of his legs for the rest of the day and night. The next day he found himself quite recovered, and he sat down to write to one of his friends an account of what had happened to him ; his letter was very long, and he wrote it without the help of glasses. Since then his paralysis has never returned. M. Leffers thinks that the same shock which restored his sight, has on the other hand injured the delicacy of his hearing.

The article from which we have extracted this case, is from the pen of M. Olmsted, professor of chemistry in the college of North Carolina.—*Phil. Mag.*

4. *Use of Mare's Milk in Tænia.*

The German physicians have lately remarked beneficial effects from mare's milk in cases of tænia. Dr. Kortum of Stalberg relates the following case in Hufeland's Journal. A lady between thirty and forty years of age had long suffered from tænia, and several attempts to remove it had failed, owing to the patient's great dislike to medicines, which caused every thing of this kind to be instantly rejected by vomiting. Having heard of several individuals that were cured by simply drinking fresh drawn mare's milk morning and evening, she resolved to give it a trial. Having an opportunity in autumn she drank two cups in the evening. Soon afterwards violent pains commenced in her bowels, and continued dreadfully severe during almost the whole night. In the morning, however, she took one cup more, after which pains in her bowels followed, but much less severe than before. In a few days a long piece of dead, and partly putrid tænia was discharged, and in a short time afterwards another piece with the narrow tapering end of the worm, and with this all the symptoms ceased. This peculiarity of mare's milk is the more remarkable, as that of the cow seems to be agreeable to the worm, and on being drank merely alleviates the symptoms.—*Philadelphia Jour.*

### 5. *Use of Nitrate of Silver in Medicine.*

The *Giornale di Fisica*, tom. xi., contains, at p. 355, a paper by the Cav. Sementini, on the use of nitrate of silver in cases of epilepsy. After remarking on the difficulty which occurs in treating such cases, and the good effects which have been observed in using the nitrate of silver, and its superiority in this respect over all other remedies, both as to the effect it produces, and the little inconvenience it causes; the Cavalier states, that to secure the good effects belonging to it, the nitrate of silver should be well triturated with a vegetable extract, in combination with which it should be given; that the first doses should be small, and the quantity gradually increased to six or eight grains, or even more, in a day: that the use should not be continued very long together; and that the patient should keep out of the action of light. The latter precaution is necessary, to prevent the discolouration of the skin, which sometimes happens after a long and copious use of this remedy. The precaution, however, only regards avoiding the meridian sun-light.

It frequently happens, in the use of this medicine, that a species of cutaneous eruption, consisting of small pustules, occurs. This may be regarded as a certain proof of the good effects of the medicine.

In the early part of this paper, the Cav. Sementini, in endeavouring to remove the impression existing against nitrate of silver, because of its poisonous qualities, remarks, that being mixed with vegetable extract, it is not really the salt, but the oxide, that is given; and, therefore, the observations of M. Orfila, on the nitrate as a poison, have nothing to do with the power of the remedy. At the same time, as an argument for using the nitrate in place of the oxide, it is remarked, that at the moment of decomposition a combination is, probably, effected between the extract and the oxide; and that actually the salt is found most efficacious.

Being assured of the use of nitrate of silver in epileptic affections, and reasoning upon its tonic effect, the Cavalier S. was induced to try its powers as a remedy in cases of paralysis. The first instance quoted is of a gilder, who, probably from the fumes of mercury, had become very paralytic. An eighth of a grain of nitrate of silver was prescribed at first, but the dose was increased every other day: by the time that three grains were taken the good effects were evident, and in twenty days more the man was perfectly restored. In another instance, every part of the body and limbs were paralyzed but the head. A small quantity was given at first, but it was increased to eight grains per day, and it effected a cure.

Three other instances are then adduced, in all of which cures were effected: and the Cavalier expresses his hopes, that, in the hands of other medical men it will be found as effective and as important as in his own.—*Philadelphia Jour.*

### 6. *Results obtained by the Use of the Iodine in the Clinico-Medical Institute of the Imperial and Royal University of Padua.*

After the effects obtained by Dr. Coindet, in the cure of the goitre, by the use of iodine, and communicated by him to the Helvetic Society, which resolved immediately on publishing his memoir, the Clinical Institute under the direction of Professor Brera, put this new remedy to the test, not indeed in the cure of goitre but to restore sanguineous assimilation, increase vascular action, and act in a particular manner on the uterine system in deficient menstruation. Five persons were subjected to these experiments. The preparation preferred was the alcoholic tincture; sometimes the iodine was united to the black oxide of manganese.

Case 1st. Maria Filippini, aged 18 years, had until within these four months past enjoyed excellent health, since then she has had suppression of the menses, by which she has been subjected to repeated spittings of blood.

Arrived at the clinical ward, and subjected to the use of the iodine,—the spitting disappeared, and she went away, her health tolerably well re-established.

Case 2d. Antonia Masa, 21 years of age, likewise wanting in her menstrual discharges for some time past: was taken with vicarious hæmoptysis. The colour of the patient was yellowish, and showed that she also was affected with diseased enlargement of the liver, the result of vascular energy.

The continued use of the iodine restored the functions of the uterus in such a manner that twice the menses flowed for six days in succession.—She is now perfectly restored to health.

Case 3d. Catherine Phillini, 22 years of age, suffering under dysentery from suppressed menstruation, was cured by the continued use of the iodine.

Case 4th. Giovanna Guerinæ, aged 16 years, pellagrous, entered the Clinical Institute with diarrhœa, reduced strength, suppression of the menses, and so emaciated that she seemed already labouring under marasmus.—The tincture of iodine restored this patient to her primitive health.

Case 5th. Maria Giacomini, 23 years of age, presented herself, complaining of prostration of strength, suppression of the menses, and in place of them a monthly loss of blood from the internal angle of the left eye.—The complexion of the patient was jaundiced, and she showed a state of preternatural assimilation in the greater number of the organic tissues, by a defect of vascular action.—When put under the use of chalybeate remedies, she almost constantly vomited.—The iodine alone was discovered to be eminently advantageous. With happy results she was then treated with the tincture of iodine, and subsequently, the same combined with the black oxide of manganese.—The palpebral hæmorrhage ceased, and true menstruation appeared in its place.—The patient gained strength and colour, but the want of iodine prevented us from continuing our observations.

It is worthy of remark, that this remedy, besides its being endowed with the property of increasing vascular action, restoring sanguification and re-establishing the ordinary sanguineous excretions, particularly from the uterine vascular system on which it would seem to exercise a direct action, excites the activity of the gastric functions, so that under its use the appetite is renewed and active, the work of digestion goes on with celerity and without inconvenience even in delicate females, and those with weak stomachs.

### 7. *Composition of Oxalic Acid.*

M. Dobereiner stated, about five years since, that oxalic acid contains no hydrogen, and that it is formed of equal volumes of oxide of carbon and carbonic acid, combined with a proportion of water. He considered this water as essential to its existence, and that, if it were taken away, the acid would be decomposed. Reflecting afterwards upon the great affinity which fuming sulphuric acid has for water, he performed the following experiment, which he has described in a pamphlet upon pneumatic chemistry :—

Five grains of dried oxalic acid, but still containing a quantity of water, were mixed with 200 grains of fuming sulphuric acid, in an apparatus for receiving gases over mercury. The oxalic acid gradually and totally disappeared, and produced 9.4 cubic inches of gas; the sulphuric acid became less fuming.

The gases, washed with ammonia, were reduced to 4.7 cubic inches, and consequently contained 4.7 cubic inches of carbonic acid. The gas which the ammonia did not absorb was oxide of carbon; for it burnt with a blue flame, and, being detonated in Volta's eudiometer with half its volume of oxygen, it produced an equal volume of the carbonic acid, without any appearance of water: the weight of carbonic acid, added to that of the oxide of carbon, represents exactly that of the anhydrous oxalic acid; and M. Dobereiner concludes that this acid contained no hydrogen; for, if it contained any, sulphurous acid should be formed; or, if the hydrogen was combined with a portion of oxygen of the oxalic acid, the carbonic gas and the oxide of carbon would be found in different proportions.

In this experiment, the sulphuric acid combines only with the water, and, in order that it may succeed, it is requisite that the sulphuric acid should be fuming, like that of Nordhassen: for common acid does not decompose oxalic acid.—*Ann. de Chimie et Phys.*

### 8. *Foreign Medical Literature.*

The Dictionnaire des Sciences Medicales is at last completed. Volumes fifty-nine and sixty contain the analytical tables of the whole work, which is thus rendered particularly useful.

Dr. Double has published the third and last volume of his "Semeiologie Generale; ou, Traité des signes et de leur valeur dans le Maladies." The first volume of this highly valuable work made its appearance in 1811. The second volume followed in 1817. It is by far the best publication of this kind extant, as well as the most extensive. The third volume contains the pathognomonic signs derived from the state of the secretions.

A work, which promises to be of considerable utility to the practitioner in the healing art, has been undertaken by M. Pelletan, jun. intitled "Dictionnaire de Chimie, generale et medicale." We have received the first volume, and augur well, from it, of the remainder.

The fourth volume of the Dictionnaire de Medecine, by Messrs. Beclard, Breschet, Cloquet, &c. has been transmitted to us. Its contents are in every respect equal, for interest and intrinsic value, to those of the former volumes. We particularly call the attention of our readers to the article *Cancer*, a master-piece of lexicographic composition. It is from the pen of M. Breschet.

### 9. *Antidote for Vegetable Poisons.*

M. Drapiez has ascertained by numerous experiments, that the fruit of *Feuillea cordifolia* is a powerful antidote against vegetable poisons. He poisoned dogs by the *Rhus Toxicodendron* (Swamp Sumac), Hemlock, and Nux Vomica. All those that were left to the poison died; but those to which the *Feuillea* was administered recovered completely, after a short illness.—*American Paper.*

### 10. *Cameleons, or Lizards of Cashau.*

Sir R. Ker Porter observed numbers of lizards and tortoises crawling along the sides of the road. Some of the former were of strange shapes, and others of unusual length; one, that he found dead, was above two feet from the nose to the tip of the tail. He remarked that these animals invariably took the colour of the ground on which each particular kind existed. If verdant, the lizard was green; if sandy, she was yellowish white; if red earth, or reddish mouldering stones, she was pink; and, if found among fragments of rock and other dusky-hued relics, she would appear of a varied brown. He leaves this fact to naturalists to explain, confessing himself totally ignorant of its secret.—*Travels,* vol. i. p. 390.

---

### MONTHLY CATALOGUE OF MEDICAL BOOKS.

### METEOROLOGICAL JOURNAL.

**By Messrs. William Harris and Co. 50, Holborn, London.**

***From April 20, 1822, to May 19, 1822.***

| | MOON. | Rain gauge | THERM. | | | BAROM. | | DE LUC'S HYG. | | WIND. | | ATMOSPHERIC VARIATION. | | |
|---|---|---|---|---|---|---|---|---|---|---|---|---|---|---|
| | | | 9 A.M. | MAX. | MIN. | 9 A.M. | 10P.M. | 9 A.M. | 10P.M. | 9 A.M. | 10P.M. | 9 A.M. | 2 P.M. | 10P.M. |
| Apr | | | | | | | | | | | | | | |
| 20 | | | 50 | 55 | 43 | 29.71 | 29.69 | 65 | 60 | WNW | WSW | Fine | Fair | |
| 21 | ● | .23 | 45 | 58 | 43 | 29.63 | 29.48 | 67 | 71 | SW | SW | Fine | | Rain |
| 22 | | .08 | 45 | 56 | 44 | 29.41 | 29.43 | 65 | 61 | WNW | WSW | Fine | | Sho'ry |
| 23 | | .10 | 47 | 53 | 45 | 29.35 | 29.54 | 67 | 62 | W var. | SSW | Rain | Sho'ry | Fair |
| 24 | | | 50 | 56 | 44 | 29.64 | 29.68 | 61 | 59 | SSW | SSW | Fine | | Cloud. |
| 25 | | .19 | 49 | 53 | 43 | 29.62 | 29.75 | 55 | 58 | SW va. | W | Rain | | Fine |
| 26 | | | 48 | 57 | 47 | 29.91 | 30.09 | 56 | 60 | WNW | WSW | Fine | | Fair |
| 27 | | .04 | 50 | 57 | 51 | 30.02 | 30.14 | 66 | 69 | SW | SW | Rain | Fine | Fair |
| 28 | ☾ | | 55 | 60 | 49 | 30.25 | 30.26 | 70 | 67 | WSW | SSW | Cloudy | Fair | Fine |
| 29 | | | 53 | 60 | 51 | 30.25 | 30.25 | 66 | 59 | SE | SE | Cloudy | Fine | |
| 30 | | | 56 | 65 | 48 | 30.26 | 30 30 | 45 | 47 | E | ESE | Fine | | |
| May | | | | | | | | | | | | | | |
| 1 | | | 53 | 60 | 45 | 30.32 | 30.26 | 50 | 58 | SE | SE | Fine | | |
| 2 | | | 49 | 60 | 44 | 30.21 | 30.08 | 48 | 55 | ESE | SE | Fine | | |
| 3 | | | 50 | 63 | 50 | 30.00 | 29.78 | 62 | 53 | ESE | E | Fine | | Cloud. |
| 4 | | | 54 | 63 | 51 | 29.60 | 29.70 | 60 | 56 | E var. | ESE | Fine | | Fair |
| 5 | | | 55 | 66 | 53 | 29.71 | 29.74 | 78 | 76 | E | E | Fine | Th.&Lig | Fair |
| 6 | ○ | | 60 | 67 | 54 | 29.78 | 29.77 | 67 | 80 | NE | NE | Foggy | Fair | Cloud. |
| 7 | | .67 | 58 | 61 | 47 | 29 79 | 29.89 | 75 | 85 | E | ENE | Cloudy | Ruin | |
| 8 | | | 50 | 57 | 43 | 29.90 | 29.94 | 71 | 68 | E var. | E var. | Cloudy | Fine | |
| 9 | | | 47 | 54 | 43 | 30.03 | 29.98 | 65 | 59 | E var. | ESE | Fine | | |
| 10 | | .21 | 46 | 57 | 43 | 29.65 | 29.41 | 73 | 76 | S | SSW | Rain | Storm | Fair |
| 11 | | | 48 | 58 | 48 | 29.68 | 29.68 | 49 | 60 | SSE | E | Fine | | Rain |
| 12 | | .18 | 46 | 50 | 46 | 29.80 | 29.86 | 66 | 72 | E | NE | Cloudy | | Sleet |
| 13 | | | 49 | 53 | 47 | 29.90 | 29.85 | 70 | 69 | ENE | ENE | Cloudy | Sho'ry | Fine |
| 14 | ☽ | | 54 | 60 | 50 | 29.90 | 29.93 | 65 | 53 | ENE | ESE | Fine | | |
| 15 | | | 60 | 68 | 53 | 29.95 | 29.97 | 50 | 53 | ESE | ESE | Fine | | |
| 16 | | | 60 | 71 | 55 | 29.97 | 29.97 | 45 | 58 | SE | SE | Fine | | |
| 17 | | | 61 | 70 | 53 | 29.89 | 29.94 | 53 | 57 | NW | SE | Fine | | |
| 18 | | | 59 | 69 | 53 | 30.03 | 30.00 | 50 | 50 | NE | SE | Fine | | |
| 19 | | | 61 | 75 | 56 | 30.00 | 30.00 | 51 | 53 | E | SE | Fine | | |

The quantity of rain during the month of April, was 2 inches and 12-100ths.

